# Memoirs of a Few Fundamental Points of Dental Medicine, Considered in Its Application to Hygiene and Therapeutics

**Published:** 1853-10

**Authors:** A. F. Talma, C. A. Du Bouchet

**Affiliations:** Dentist to the King of Belgium, &c. &c. &c.


### ARTICLE XVI.

*Memoirs of a few Fundamental Points of Dental Medicine, considered in its Application to Hygiene and Therapeutics.* By A. F. TALMA, M. D., Dentist to the King of Belgium, &c. &c. &c. Translated by C. A. DU BOUCHET, M. D., D. D. S. First Series. Brussels, 1852.

THE permeability of the teeth and the persistence in their tissues of a circulation, doubtless feeble and obscure, are facts which physiological observations and direct experiments positively establish beyond a doubt. These observations and experiments strengthen the inductions deduced from the examination of their anatomical structure. M. Muller has seen ink rising by capillary attraction in the tubuli of the teeth of the horse. M. Serres has observed, in several preparations, blood globules choking up the canaliculæ of the dental cavity and forming three distinct striæ. Contrarily to the experiments of Hunter, on the nutrition of young animals, M. Flourens has demonstrated that by the use of madder, it is not only that portion of the teeth, the formation of which takes place while the animals are subjected to this treatment, which acquires the peculiar hue, but likewise the parts of the organ previously existing. In some cases the teeth, even their enamel, assume a manifest sanguineous tint, and receive a coloration which can be due only to the repletion of their vessels. It is perfectly recollected that during the cholera epidemic in 1832, several surgeons, and among others M. Begin, have presented to the Royal Academy of Medicine of Paris, teeth which, as well as the other parts of the skeleton, presented a cyanosed tint analogous to that of the skin. The same observation has been made again, during the last epidemic of 1849.

After recalling the opinion of Beclard, who, although not willing to acknowledge any vascular communication between the dental pulp and the ivory of the crown, still admitted that this latter substance is continually receiving from the pulp a fluid by imbibition. Messrs. Desirabode leave this part of the question undecided. As to the ivory of the roots, do they add: we think that its texture, the organic action and the morbid alterations developing themselves in the roots, although not making evident, vessels continuous with those of the general organism, authorize at least to consider the question as settled in favor of their presence. But upon what principles, derived either from the evolution or structure of the teeth could the learned authors we have just quoted, establish such a distinction between the ivory of the root and that of the crown? No line of demarkation between the two parts is found to justify such an opinion, and that which is applicable to the one must also be applicable to the other.

It has been impossible for me to allow the interesting labors of so many observers to be published, without endeavoring to ascertain their correctness. A long time previous, besides, I had been led, by the alterations of dental tissues, to conclusions analogous to such anatomical examinations and physiological principles have permitted us to arrive at, and certainly this very circumstance of similar results in pursuing such dissimilar roads, is highly gratifying.

With the assistance of a good magnifying glass, it is easy to see on the surface of the eroded portion of a carious tooth a cellulo-vascular layer, reddish, brown or black, granulated irregular, and exhibiting the appearance of the fungous flesh of certain ulcers of bad character. If, after having made a section perpendicularly to the ulcerated surface, we examine the plan of the section, it is easy to perceive in the invaded portions, alterations entirely vital; upon the points nearest to the ulcer, the enamel or the bony structure are softened, friable, of a brown or black color; as it dips into the substance of the tooth, the tint becomes less deep, and is, finally, through gradual shades of a more or less yellow cast, lost into the normal color. Upon this morbid field, more or less extensive, the eye, aided by the magnifying glass, distinguishes lineaments, striæ, which demonstrate the presence of canaliculæ, or of vessels much enlarged. It seems that in the teeth as in the caries of bones, especially in the denser portion of the latter organs, the increased vascularity precedes the softening and prepares for the destruction of the tissues, whose solid molecules are disintegrated or absorbed by the living fabric.

These phenomena become more apparent still, most incontestable, when we cut the dental substance, starting from the carious portion into lamellæ thin enough to render them very transparent, and permit us to examine them in holding them up to the light. Then the graduation of the tints, the diminution of the proportions of the solid or saline portion of the organ, the preponderance on the contrary of the cellular, tubulated or vascular portion become facts of a palpable evidence.

I could easily multiply these examples, in going over the series of the affections of a vital origin, such as exostosis, erosion, the softening of the dental substance, but they will come in better order when I have occasion to treat of those diseases especially.

If I have so strenuously insisted upon the anatomical, physiological, and pathological facts, demonstrating the vitality of the teeth, it is because this point is of an importance of the first order. This vitality is, in fact, the key to all dental medicine; it *alone* can connect it with general medicine, by links which reason and experience do not disapprove of. Let this vitality be considered as not existing, and the art of the dentist is no longer but a mechanical affair, more or less ingenious, more or less complicated, which will no doubt require a special dexterity, but which will no longer have but a distant relation to medicine. Let, on the contrary, this vitality be demonstrated, a light suddenly illuminates the hygiene, the pathology and therapeutics of the dental apparatus, and they immediately rest upon rational and scientific bases, from which they cannot be removed.

If the teeth were in organic bodies, as has been advanced by eminent men, (rather naturalists than physicians,) how could we connect their organization and diseases with the general constitution of individuals? How could we account for the influence exerted over them by atmospheric variations, and the thousand circumstances of regimen and morbid causes in the midst of which we live? Once developed, would not the teeth, according to that hypothesis, be exclusively submitted alike those of our artificial sets, merely to those mechanical or chemical causes of destruction capable of wearing out or breaking them, or decomposing their substance? By what rules, drawn from physiology and pathology, would it be possible to prescribe those means of treatment with the aid of which we however succeed in modifying, preserving, and even sometimes restoring to their normal condition, organs which but for that vitality would only be in the mouth of patients, carved ivory or fragments of porcelain? It is by starting from this fundamental principle of the vitality of the teeth, throughout the entire period of their duration, that we are able to distinguish, amid so many means proposed for the cure of their diseases, those which are really useful from the others, the use of which might be injurious, and even often dangerous.

II.—*General Importance of the Care and Attention bestowed on the Teeth.*—It is much to be wished that, in certain countries, where the diseases of the teeth are unfortunately so widely spread and so often assume the worst type, each one should feel the necessity for rational and constant attention, as well as the opportune assistance of the man of the art to watch, and, if needed, direct the labors of nature in young subjects, until the epoch of the entire consolidation of the dental apparatus.

As I have already stated in one of my previous publications, the medicine applied to the study and treatment of the affections of the mouth and teeth, reposes upon the same principles as general medicine. The latter gives to every one advice based upon experience, to preserve health; but when illness comes, when important organs begin to suffer, the assistance of a skilful physician becomes necessary. It is even prudent, as has been laid down by sound writers, to speak from time to time about one's health and ask for advice, especially at those critical epochs of the organism when the body undergoes certain modifications, more or less intense, which dispose to disturbances of functions and disease. How many families have lost the objects of their fondest hopes, only by their too late recourse to medical advice? How many others are indebted for the happiness of still preserving their offspring, to the careful solicitude with which they have constantly surrounded them.

These reflections are in every respect applicable to the diseases of the mouth. The profession may express and diffuse, for the preservation of this important part of the economy, general principles easy to be followed and of incontestable utility; but when, notwithstanding the observance of these precepts, or in consequence of paying no regard to them, diseases develop themselves, the physician alone can discover those diseases in their origin and oppose to them appropriate means, before they have determined unretrievable alterations. The dentist alone can, during the labor of the eruption and arrangement of the teeth, prevent the disagreeable or hurtful arrangements which these organs are liable to assume, or remedy it at the outset, and correct anomalies which, at a later period, could no longer be corrected.

These are truths of universal application, which I have always deemed a duty to inculcate in the minds and customs, in the place of the careless barbarity, or the absurd prejudices too generally met with among communities.

Childhood, old age, trades, professions, the organs of locomotion, respiration, and other more important functions, are the object of rules and precepts which enlightened persons study and observe. Why, then, should the mouth be neglected? Why, during youth, and even during life, should we not entertain the same anxiety concerning the dental apparatus, as we do for other parts less exposed to the sight, and often less important to health.

III.—*Cares Relative to the First Dentition.*—As early as the first age in life, the mouth, and particularly the dental system, need special attention. If a few physicians have exaggerated the dangers attached to the labors of the first dentition, it is to be feared that others, considering this labor as foreign in the greater number of cases, to the diseases of children, should fall in the opposite exaggeration far more dangerous, inasmuch as it tends to cause to neglect useful precautions, and lead away from the research of important phenomena. Whether dentition be the primitive cause, or only the predisposing cause of the diseases developed, pending its entire duration—whether it be their termination, or complicate and accompany them only, it cannot, in either case, ever be considered as an indifferent circumstance, and we must make full allowance for the share of action it may exert over the progress and intensity of accidents. Universal opinion is in harmony with this precept, and the greater mortality among children at this time of life than at any other period, renders it imperative not to neglect it.

It is not my purpose to expatiate at length upon the multifarious affections which may result from the various sympathetic reactions of the dental system, set in action upon the cutaneous, respiratory, digestive apparatus, and especially the nervous system. These developments appertain to general medicine. It will be sufficient for me to say, that, from the third month to the middle of the third year, a period, the commencement of which corresponds with the first efforts of the eruption of the incisors, and which terminates by the appearance of the second molars; that, during that period, do I say, every time an affection of some importance shall arise spontaneously in the child, with phenomena ever so little insidious, the first care shall be to ascertain the progress of the dentition, to examine the mouth, and take due notice of the condition of the alveolar ridges. It must be borne in mind, that the eruption of the teeth is the more difficult, the more susceptible to be accompanied with grave accidents, the larger the teeth are endeavoring to force their way out; little felt when incisors are in question, but much aggravated and painful when canines and molars. We must also remember, that the eruption of several teeth at the same time, is liable to cause more intense reactions.

Whether the examination of the mouth displays in the heat of that cavity, the abundance of saliva, the tumefaction, the redness, the softening of some points of the gums, the traces of a painful labor to which may be traced the morbid phenomena just enumerated; whether the complete inertia and the normal condition of the buccal tissues remove any idea of that kind, in both cases, the practitioner will have done much for the etiology as well as the diagnosis and treatment of the disease he intends to contend with.

When the cause of the disturbance seems to lay in the difficult eruption of the teeth, or when this circumstance adds itself to the accidents and aggravates them, we should remedy it. Mild gargles, soft bodies, wet with slightly sweetened mucilage, will be held to the mouth, or given to the child usually fond of biting. If the gums are much swollen, of a deep or lividinous red; if the fever, heat, and restlessness are considerable; if the little patient is plethoric, one or two leeches will be applied with much advantage to the angle of the jaws, and revulsives in the meanwhile applied to the feet. A few slight punctures, made with the point of a lancet on the gums, will have a tendency to deplete them more directly. If aphthæ are present, they should be washed with a mucilaginous decoction sweetened with honey, and suitably dosed with hydrochloric acid.

These means usually suffice, if not to restore quiet, at least to allay local accidents, and enable nature to finish her task. In more grave cases, and particularly when nervous phenomena of somnolence or convulsion place the life of the patient in jeopardy, it may become necessary to perform a more serious operation. I mean the incision of the gum over the teeth, the eruption of which is too tardy, or meets in the density of the covering tissues an unusual difficulty. This operation should never be performed inconsiderably. Although trifling, it frightens the parents, causes pain, may expose, if the hand of the operator is not steady, to wound some part of the mouth, and, finally, does not seem to have at all times been practiced without inconvenience, either for the gums, which have sometimes suppurated, or for the teeth, the premature decay of which has been attributed to lancing.

Before having recourse to it, it is necessary on one part, that serious morbid accidents should demand it, and require that the effort of eruption, the origin or aggravating complication of these accidents, be facilitated or shortened. On the other part, it is equally necessary that the tumefaction, redness, pain in the gums, should announce that the local labor has already made considerable progress, and that the tooth seems ready to appear. When these two conditions are met with, the incision of the gum acts as a complete sedative, accompanied with local depletion, which procures at once the relaxation and disengorgement of the tissues. * * * *

A very important observation must be added to the preceding. Far more frequently than practitioners are willing to admit it, the accidents attributed specially to first dentition, are the result of the preparatory labor of the second dentition; hidden, deep labor, displayed by anatomical inspection, shaking the whole maxillary apparatus. The development of the crowns of the permanent teeth, coincides, in fact, for the greater part, with the successive eruption of the deciduous teeth. If, as happens with certain subjects, the second teeth are larger than allowable by the condition of the jaws, they will exert upon the latter a considerable pressure, will determine the distension of the cavities enclosing them, and the dull irritation of all the surrounding organic elements. This fact is produced particularly when the first teeth are small, whilst the permanent ones are preparing on a much larger scale. * * *
It will be seen, that it becomes very difficult to distinguish in complicated morbid disturbances, what may belong to each of these two orders of disturbances, whose action is sometimes isolated and sometimes united. When the eruption of deciduous teeth is the only cause of disturbance, the incision of the gum is usually efficacious; without proving hurtful, it remains insufficient in the cases in which the development of the permanent teeth is the cause of the accidents.

I have remarked, that when the permanent teeth are in bearing with the jaws, and meet with no obstacle in their growth, the deciduous teeth usually appear without any difficulty, inducing no bad symptoms, and often unnoticed, unless the child be of a very susceptible diathesis.

These circumstances perfectly explain the different opinions of many physicians and dentists, relatively to an operation, in itself deprived of danger, but whose success depends upon fugitive conditions, requiring much experience and skill to be properly appreciated. I must repeat, that in cases of grave accidents, of which dentition may be the origin or complication, in order to derive from the incision of the gums all the benefits we should expect, it is necessary, that the gum over the tooth or teeth in process of eruption, be raised, reddened, tumefied, hot, tender to the touch. In this condition, the tooth is near and ripe, as Duges correctly expresses. If the gum is softened, stretched, and exhibits a white spot, a sort of pellicle, this appearance indicates that it is almost completely absorbed or worn off, and that under that pellicle exists a projecting portion of the dental crown.

In other words, it is necessary that the labor should be *localised;* that one or several teeth be very distinctly prominent, that the gum be particularly inflamed, and raised upon the elevated points; if, on the contrary, the gums are vaguely tumefied and inflamed, especially at their base, we must infer that this condition of things depends upon deep-seated disturbances, cause by the development of the permanent teeth, and that incisions would prove useless.

Finally, it sometimes happens, that the gum is pierced upon a point, and the tooth makes its appearance, but having reached this degree, especially as regards molar teeth, the labor becomes stationary, and requires to be terminated by the intervention of the art. This indication presents itself in many subjects when cutting their dens sapientiæ. The operation itself is one of the simplest. The instrument should be guided with precision, by the fore-finger of the left hand; the nail marks the place where the incision should be made, and limits its extent. The simple section, parallel to the alveolar ridge, is only adapted to incisors. Canines and molars require a crucial incision, and even, if possible, the angles should be raised with the instrument.

After the operation, the flow of blood, always useful in such cases, should be promoted by means of gargles with lukewarm mucilaginous water, and it is proper to continue the use of the means of treatment previously employed. The improvement is, in many cases, almost instantaneous, and the most formidable accidents have been known to disappear in a few hours as if by enchantment.

I must here recall that accidents determined by dentition, or complicating it, are the more frequent and the more grave, as the subjects are the more weakly, irritable, unhealthy; badly ventilated dwellings, uncleanliness, bad food, improperly directed artificial lactation, manifestly predispose to them. We must then, as far as possible, in order to prevent them, surround children with well-directed hygienic cares, among which, living in the open air, suitable clothing, frequent tepid baths, cutaneous frictions with flannel, and especially proper alimentation hold the first rank.

IV.—*Cares relating to the Second Dentition.*—§ 1. *Normal Phenomena.*—In the preceding article, I have noticed that the development of the crowns of the permanent teeth, coincides, in a great part, with the successive eruption of the temporary or milk teeth. Lodged in distinct cavities, beneath and back of the first teeth, the permanent ones enlarge the bony cells which contain them, destroys the partitions which separate them from the primitive corresponding teeth, act upon the roots of the latter, wear them out, shake them, and contribute to determine their fall.

There can be no doubt, that this admirable phenomena of the removal of the deciduous teeth is as vital as mechanical. These teeth become loose, detached and fall, in conseqnence of the same law which separates the fruit from the tree. Even if the tooth of replacement should not exist, or deviating in its situation, it should not touch the root of the one it is corresponding to, the result would be the same, excepting as to time. The obliteration of their nutritious vessels, the atrophy of their means of union with the alveolar walls, the loss of life, in a word, within or without, is, in fact, sufficient to isolate milk teeth, render them foreign to antagonism and hasten their fall.

The replacement of these teeth takes place nearly in the same order as their primitive eruption. The first dentition being completed from six to seven years, by the eruption of the four first large or permanent molars; the second begins with the lower central incisors, which are shed from seven to eight years. A short time after, the superior central incisors appear; then from eight to nine years, the lateral incisors, still commencing by the lower jaw; from nine to ten years, the four first small molars or bicuspids; from ten to twelve, the canines and the second bicuspids; from twelve to fourteen, the second large molars, and finally, from eighteen to twenty-five, the last teeth of this category, or wisdom teeth.

The second dentition is seldom accompanied, like the first, with serious disorders, or intense reactions upon the principal organs of the economy. The labor of eruption is progressing with more slowness than that which preceded it; when it takes place, the subjects are more developed, their functions already have a more permanent equilibrium; finally, these teeth appearing almost as soon as their predecessors have fallen off, have not to overcome the resistance of the tissues of the gums. It is generally only on the occasion of new permanent teeth, such as large molars, or more particularly wisdom teeth, or anomalies in the direction of the other teeth, that we see accidents, local and general, which it may become necessary to remedy.

If, then, the epoch at which begins the shedding of the twenty deciduous teeth, generally constitutes a period critical for children, it is less on account of the labor of which the dental apparatus is the seat at that time, than in consequence of the new enlargement assumed by all the parts of the organism, manifested by this very labor.

But if it is so concerning general health and preservation of life, it is not the same as regards the special disposition and arrangement of the maxillary apparatus. During the second dentition, it acquires its definitive conformation, its more or less irregular arrangement. During that time, results are produced, which shall be, in many cases, lasting as life. This period, then, is of an extreme importance respecting the conformation of the mouth, and during all its progress, the dentist should be called to exercise all his skill and care. When the second dentition is completed, the teeth symmetrically arranged on the alveolar ridges of both jaws, should form two regular lines, representing the two halves of an ovoid, of which the superior arch constitutes the larger half, and the inferior the smaller one. The convexity of the former brought near to the latter, is more flaring out, more rounded. The teeth which it supports, are more voluminous and slightly inclined forward; whence it results that they cover the inferior ones, and gently overlap them. The direct antagonism only begins at the molar teeth. The length and projection within and without, should be equal over all the maxillary line; each tooth should present a smooth and polished surface, perfectly parallel to the curved surface which it fills up, so as to contribute to the harmony of the whole.

The texture of the teeth includes numerous varieties as regards appearance and solidity. Several characteristics resulting from these differences, are hereditary or transmissable from parents to children; others appear to be proper to certain countries; some are attributable to special hygienic habits, to the use of certain substances, such as tobacco, betel, or that of aliments or drinks, variable in composition, density, temperature, etc.; finally, the individual regimen, and the general constitution of the subjects, exercise over the texture of the teeth an incontestable influence. Teeth of a bluish, milky-white, as if transparent, are generally delicate, irritable, little resisting; they are met with, usually, in lymphatic, nervous, delicate subjects, predisposed to strumous affections. The most solid teeth are of an opaque yellowish-white, approximating the color of the bones. They are one of the characteristics of a robust, bilious, or sanguine temperament, and are usually connected with activity and power of the digestive organs. Women generally have teeth smaller, and of a more brilliant white, than men.

The perfection of the structure of teeth, and their resistance to the various causes of diseases and destruction to which they are exposed, depend less on the proportions of the calcareous salts entering into their composition, than on the good constitution of their organic element. From the analysis made by M. Lassaigne, milk teeth contain more phosphate of lime than the permanent teeth, (67 per cent. to 61 per cent.,) and are, nevertheless, less durable. In the skeleton, the bones, in taking up saline principles, become more friable, and more easily fractured. If we find in the structure of the teeth, and in that of the bony system, the impress of the vital energies of individuals, this impress is manifestly connected with the organic part of the tissue, presiding to the nutrition of the organ, not with its inert constituents, whose abundance, mollicular arrangement and cohesion are subordinate to the conditions of life.

§ 2. *Accidents.*—In consequence of this incontestable fact, that permanent teeth are formed and developed as early as the first period of life, it is evident that the observance or neglect of the rules of hygiene during these periods, the manifestations or absence of maladies in young subjects, must exert a notable influence upon the qualities of the teeth as upon the whole of the living economy. This influence had not escaped the father of medicine: these judicious remarks have been confirmed by the best modern observers, especially M. Mohon, who has extended and defined them. Struck with the importance of this subject, both as regards physiology and appreciation, it has been for a long time the object of my researches in public institutions, as well as in my private practice. My observations, compared with those of others, recently published, have led me to some results not entirely devoid of interest.

If a child, between birth and the age of twelve or eighteen months, is affected with one of those serious disorders which strongly shake the constitution, such as convulsions, cerebral fever, softening of the osseous tissue, the four permanent incisors, the canines, and the first large molars, will most usually exhibit on their surface, transverse lines, more or less deep, asperities, small pits, with dark stains, and their free edge will remain denticulated, sharp. Sometimes they will be found yellowish, slender, stunted. The situation of these alterations at the same horizontal height, on several of the teeth effected, indicates that the latter have simultaneously undergone the effect of the cause modifying their structure. If the disease liable to produce these effects manifests itself at a later period, say from two to four years, the bicuspids and the second molars will be liable to be impaired; the teeth precedingly mentioned having their crowns formed, remain, on the contrary, in their normal condition.

The interference attributable to the diseases of children in the organization of the teeth, is not always revealed under the same appearance. Sometimes, in the place of striated lines or black punctures, dividing the crowns, we find irregular stains of an opaque or yellowish-white, which, without impairing the polish of the enamel, give it an unpleasant appearance. Finally, the alteration of the dental structure may escape observation, whether it involves the roots or the dentine exclusively. Thus, in many subjects, teeth symmetrically arranged at the two sides of the median line, and in both jaws, are attacked at the same epochs of life by morbid affections of similar nature, and are destroyed in consequence of indentical affections, whilst the remainder of the teeth preserve their normal condition, and frequently remain healthy in the mouth to a far advanced age. These phenomena indicate a common action, probably exerted at the same time on the organs which experience the same fate, whilst the neighboring organs, of a same nature, but of which the development has not been disturbed, preserve their integrity.

As I have already noticed, the second dentition is always less laborious than the first. We can, however, not unfrequently observe, during its progress, some more or less serious accidents. The gums often become tumefied, red, painful, and in some subjects this turgescence is propagated to the different parts of the mouth, and even to the pharynx. We also observe in a few children, an abundant flow of saliva, numerous aphthæ, or even small ulcers, produced by the softening of the more inflamed points. To these local symptoms are added, when they are intense, heat in the mouth, thirst, restlessness, sometimes fever, and more rarely, nervous accidents. During the entire process, children are, moreover, exposed to various irritations, such as sore eyes, headache, general, or limited to one side of the head, bran-like eruptions on the face or scalp, engorgement of the lymphatic ganglions, submaxillary parotidian, and cervical. In lymphatic subjects these transient glandular inflammations have a decided tendency to become permanent, and constitute the origin of scrofulous tumors.

In a great number of subjects we notice, towards the age of ten to twelve years, various disturbances of digestion, a general and unaccountable malaise, paleness, and other indispositions, which we can attribute only to the preparatory labor for the eruption of the bicuspids, the second molars, or the canine teeth. These phenomena are the more marked, as those teeth are larger, and consequently exert a more considerable dilating pressure on the alveolar walls.

The eruption of the wisdom teeth is more particularly difficult, and very often painful. These teeth do not always find room enough to place themselves. Pressed between the molars and the base of the coronoid apophyni, the wisdom teeth of the lower jaw often cause dull, deep pains, shooting to the ear, to the temporel region, to the entire side of the face, and having irregular exacerbations, which, according to the age of the patients and their antecedents, have often been taken for rheumatic or neuralgic affections. Those of the upper jaw more readily deviate backward, raise up the intermaxillary commissure, and more commonly cause pain in the maxillary sinus, in the ear, and in some subjects obstinate opthalmia, the cause of which frequently remains unknown. Finally, in certain cases, we observe persisting pains in the face, sick headache, and especially a persisting seclusion of the jaws, to a point rendering the introduction and mastication of food very difficult.

In regard to the local accidents, I must add that the irritation kept up by the labor of volution of the permanent teeth, and especially the wisdom teeth, is often accompanied with swellings, followed by abscess opening along the maxillary ridge, or even outside the mouth at some distance from the seat of disease.

These openings remain fistulous as long as the teeth which keep them up is not extracted. Their origin may be the more difficult to ascertain, as in many cases the patient feels and complains of no pains in the part affected. I have seen fistulæ of this kind which had afflicted the patient for eighteen months, or even several years, the cure of which followed in a few days the extraction of the teeth which caused them.

All the affections which I have just briefly reviewed, have their origin in the exaggerated irradiations of dental congestion toward the adjacent parts, and their diagnosis is often surrounded with much difficulty and uncertainty. In the absence of local alterations, enlightening him at once, the practitioner should be guided by the attentive observation of the morbid phenomena, making due allowance for their origin, obstinacy and succession. The accidents, whatever be their nature and their seat, have almost always a characteristic of their having manifested themselves without an apparent cause, externally or internally, their resisting the usual means of therapeutics, their appearance or disappearance without a plausible reason, according to the increase or decrease of energy assumed by the dental evolutions.

In the greater number of subjects the eruption of permanent teeth, and more particularly that of the wisdom teeth, is accomplished only by a series of efforts, which nature seems to abandon to a certain degree, to resume them afterwards with the same train of morbid phenomena, the duration of which is prolonged sometimes during several years. These sorts of fits, reproduced at variable intervals, almost always obscure the diagnosis, and tend to lead the practitioner into error, until the more attentive observations of these phenomena indicates at last the nature of the disorder.

The indications to remedy the accidents of irritation and phlogous just mentioned, are generally simple and efficacious. They do not essentially differ from those claimed, under the same circumstances, by the first dentitions. We must still employ assuaging means, local sedatives; in some cases determine gargles, punctures on the alveolar ridges, capillary depletion by means of leeches; and finally, when the gums remain raised up or imperfectly perforated, the crucial incision will destroy the tensions, and cause the pain to cease almost instantaneously.

It results from my numerous observations, that the operation of lancing the gums in the cases requiring it, is the more efficacious during the second dentition than during the first. When we have to facilitate the eruption of permanent teeth, the disposition of the parts and the local phenomena render, almost always, the indications precise, and the age of the patient permits them to lend themselves to the operation, as well as to bear it better.

In some cases, when the wisdom tooth does not find at the extremity of the arch, sufficient room, it becomes absolutely necessary to remove it, or to create for it the room it requires, by extracting the molar tooth immediately preceding it; but this operation being one of those which may be necessitated for the proper arrangement of the teeth, we shall again refer to it.

As I have observed, in speaking of the first dentition, it may happen that the accidents of the second be determined by the simultaneous eruption of a certain number of teeth, and by the too great amount of labor resulting from it. One of the most interesting cases of this kind is related by M. Delabarre. He states that a female child, eight years of age, of a very nervous-sanguine temperament, complained of pain in several deciduous molars. Soon a slight cough, the dilatations of the pupil, the increased irritation, loss of sleep, caused recourse to be had to the physician. Notwithstanding the judicious exhibition of sedatives, the accidents persisted without its being possible to recognize any characterized disorder. Later, febrile exacerbations appeared during the day, and delirium at night. During the intermissions, pain in the teeth.

M. Delabarre, called in his turn, thought that the accidents were caused by the too great promptitude with which the eruption of the permanent teeth was taking place. In fact, the right central, the two lateral incisors, and four bicuspids displayed their crowns at the opening of the appendices of the dental matrices. The absorption of the roots of the deciduous teeth had not had time to take place, and several of these teeth were pushed forward by the new ones. The gums were red and sensitive.

Two of the teeth, which appeared to form the greater obstacle, were extracted, and on the same day the fever ceased. The difficulty seemed to be removed; but four days after, the primitive accidents reappeared, and it was necessary to extract the two deciduous molars opposing the eruption of the bicuspids. Then only did all the symptoms diminish, and in a few days convalescence was perfect.

I have met in my own practice with a tolerable number of cases analogous to that of M. Delabarre, and by relieving the mouth of the too persisting deciduous teeth, have equally seen the accidents cease promptly. It must be remarked, however, that the extraction of these teeth should take place only when we have the certainty of the presence and forward development of the subjacent teeth; by a premature extraction, we would expose ourselves to wound or destroy the others, and even destroy their germ, as has been observed after similar operations performed on very young subjects; the loss occasioned by such malpractice is thus irretrievable.—*Dental News Letter.*

